# Relations of Diseases of the Eye to General Diseases

**Published:** 1895-10

**Authors:** 


RELATIONS OF DISEASES OF THE EYE TO GENERAL DISEASES. By Max Knies, Professor Extraordinary at the University of Freiburg. Forming a Supplementary Volume to every Manual and Text-book of Practical Medicine and Ophthalmology. Edited by Henry D. Noyes, A. M., M. D., Professor of Ophthalmology and Otology in Bellevue Hospital Medical College, etc., etc. Octavo, 470 pages, illustrated, extra muslin, price, $4.25.

In its purpose, as in its manner of presentation, this work of Professor Knies is admirable, distinctly filling a want in medical literatute in the English tongue—a saying exceedingly rarely applicable to the books of the present day. Much the same information, it is true, may be gleaned in a few treatises on ophthalmology, and here and there in systematic treatises upon neurology and general medicine; but there is nothing of recent date presenting this character and amount of information as concisely, clearly and fully. It gives the general practitioner easy recourse to material relating to ocular symptoms of practical value to him in the recognition and care of the diseases constantly presenting themselves before him, and in numerous instances should suggest new paths toward diagnosis. The book was prepared as a second part to the author's work upon diseases of the eye, and is published separately, in order to give it a wider field in the profession, it being essentially a work for the general practitioner. It is particularly full and satisfactory in its descriptions and discussions of the ocular conditions met in the various diseases of the nervous system, as might be expected; but the wide range of ocular complications and symptoms is well and thoroughly described in the separate chapters upon the ophthalmic states met in diseases of the skin, of the digestive organs, respiratory organs, circulatory organs, urinary organs, sexual organs, poisoning and infectious diseases, and in constitutional diseases. The work is for the most part a book of reference, rather than intended for continuous reading; but in many portions, particularly that devoted to the discussion of the anatomical and pathological relations of the nervous system, there is met a presentation of facts in a light that will well repay the closest reading.

As claimed by Professor Noyes, the editor of the American edition, the book is indeed a cross-index between general medicine and ophthalmology, and as such fulfills an excellent purpose. A. J. S.

